# A Novel Evaluation Method for Detecting Defects of the Bonded Orthodontic Bracket-Tooth Interface

**DOI:** 10.1155/2021/6634595

**Published:** 2021-03-15

**Authors:** Mona Aly Abbassy, Turki A. Bakhsh, Ahmed Samir Bakry

**Affiliations:** ^1^Department of Orthodontics, Faculty of Dentistry, King Abdulaziz University, Jeddah, Saudi Arabia; ^2^Alexandria University, Alexandria, Egypt; ^3^Operative and Esthetic Dentistry Department, Faculty of Dentistry, King Abdulaziz University, Jeddah, Saudi Arabia; ^4^Conservative Dentistry Department, Faculty of Dentistry, Alexandria University, Alexandria, Egypt

## Abstract

**Background:**

Orthodontic patients are at high risk to develop caries. This study is introducing a clinical method detecting interfacial defects between ceramic brackets and enamel utilizing optical coherent tomography in addition to using the nanoleakage expression in vitro test.

**Methods:**

Transbond XT primer and moisture insensitive primer (MIP) were bonded to 75 human premolar enamel surfaces and divided into (XTD), (MIPD), and (MIPW) groups. The (XTD) and (MIPD) groups had ceramic brackets bonded to dry enamel surfaces using TransBond and moisture insensitive primers, respectively, while the (MIPW) samples were bonded to moist enamel using moisture insensitive primer. All specimens were examined under crosspolarization optical coherence tomography. Debonding forces of the brackets to 45 teeth (15 teeth/group). 30 bonded specimens (15 specimens/group) were cross-sectioned to detect the nanoleakage expression using scanning electron microscope equipped with energy-dispersive spectroscopy (SEM/EDS). The degree of conversion of the specimens in the experimental groups was tested using attenuated total reflectance Fourier transform infrared spectroscopy (FTIR/ATR).

**Results:**

Optical coherence tomography detected the interfacial defects between the ceramic brackets and tooth structure. One way ANOVA showed that (XTD) and (MIPD) groups recorded significantly higher bond strength values and less nanoleakage expression when compared to MIPW (*p* > 0.05).

**Conclusions:**

Optical coherence tomography can be utilized to detect interfacial adhesive-tooth defects. Dry enamel surfaces improve the quality of the enamel/primer interface (200 words).

## 1. Introduction

One of the chief goals of orthodontic treatment is to achieve a stable interface between orthodontic brackets and tooth enamel with low susceptibility to degradation over time [1]. It was previously recommended that all measures should be taken to keep the outer enamel surface intact as it contains the highest degree of mineral and mechanical properties to minimize the enamel damage during the bracket debonding phase [1]. However, the high rate of bacterial biofilm formation observed in orthodontic patients [2] exposes the bracket-resin-enamel interface to continuous acidic challenge [2]; thus, the proper sealing of the aforementioned interface is an essential element in preventing the ingress of bacterial toxins and acids under the bracket region which may lead to enamel demineralization [3]. Such lesions are extremely difficult to be controlled or remineralized because it is located under the bracket region [3]. The detection of such lesions in an early stage may allow its remineralization instead of restoring such lesions after debonding of the orthodontic brackets, which agree with the principals of minimal interventions in modern dentistry that advocate remineralization rather than restoration of enamel and dentin lesions [4, 5].

Optical coherence tomography (OCT) is an imaging system that is utilized in medical fields to diagnose various lesions, especially in ophthalmology [6]. It provides volumetric and cross-sectional images safely, noninvasively, and nondestructively [6]. Previous research showed encouraging results for utilizing Swept-source OCT for detecting white spot demineralization lesions around orthodontic brackets [6]. However, the ability of evaluation of adhesive interfacial defects under ceramic orthodontic brackets is still an untested potential for the OCT technology [6].

Moreover, bonding of brackets to malaligned teeth, partially or completely unerupted teeth, is a challenge faced by many orthodontists that render the field isolation prior to bonding an extremely difficult process. This may negatively affect the brackets' bond strength [7] causing early and repeated debonding of the orthodontic brackets. This challenging environment renders the sealing ability of the orthodontic bonding resin a questionable matter.

The nanoleakage examination can be a suitable candidate for accurate determination of the sealing properties of orthodontic resins [8]. Nanoleakage studies are different from microleakage because nanoleakage can give a clear image for any porosity or defects resulting from deficiency in resin polymerization or infiltration within the substrate that it is bonded to [1, 8].

Orthodontic bonding systems capable of polymerizing in challenging the oral environment including enamel surfaces that are difficult to isolate prior to bonding were introduced in the market, such as moisture insensitive primer (MIP) [9–11]. However, controversial results were obtained regarding its bond strength [7, 9–12], and there is scant information regarding the efficiency of this primer in sealing the resin-enamel interface of the orthodontic brackets under moist conditions.

The current experiments aimed at examining the capability of OCT technology to detect interfacial defects under orthodontic brackets and correlate the obtained results to nanoleakage, debonding, and degree of conversion results.

The null hypotheses adopted in the current research are that OCT will not be able to detect any interfacial defects under the ceramic orthodontic brackets, (MIP) will not seal the enamel-resin interface under moist condition, and will score low bond strength values when bonded to moist enamel.

## 2. Materials and Methods

### 2.1. Specimen Preparation

Extracted human sound premolar teeth that were extracted for orthodontic reasons were collected from the oral surgery department after obtaining the permission of the ethical committee of the faculty 055-02-19. The teeth were hand scaled from any calculus or soft tissues. The teeth were stored in 0.1% thymol until the start of the experiment according to the guidelines approved by University and in accordance with the principles of the Declaration of Helsinki and its later amendments or comparable ethical standards. The number of specimens assigned to each group was adopted according to previous literature and according to the 80% power of test. Randomization of the specimens was done using a computer program (Excel 2007, Microsoft, Redmond, WA, USA). All teeth were examined by light microscope to exclude any teeth having cracks, demineralization, or any defects. Intra and interexaminers calibrations were conducted before the actual recording of the obtained results.

### 2.2. Materials Used in This Study

Two different adhesive primers were used on the enamel surface: Transbond XT primer (XTP; 3 M ESPE, USA) and Transbond MIP Moisture Insensitive Primer (MIP; 3 M ESPE, USA). Transbond PLUS color change adhesive (3 M ESPE, USA) was used to bond the orthodontic ceramic brackets (Victory series, 3 M Unitek, USA). The lot number and chemical composition of each material are according to the information provided by the manufacturers (Table 1).

### 2.3. Bonding of Orthodontic Brackets

**Figure 1:**
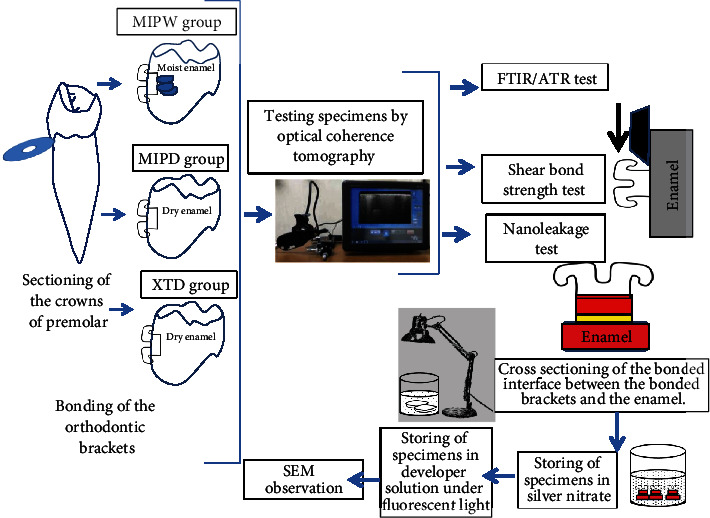
Summary of experimental procedures.

Forty five caries-free human premolar teeth extracted due to orthodontic reasons were used in this study. The teeth were divided into three groups with the buccal surface of each group being treated with XTP group or MIPD group in which XTP and MIP primer adhesives were applied on the dry enamel surface according to the manufacturer instructions, while teeth in the MIPW group 0.02 ml distilled water [12] was added to the surface, and the water was not removed. Transbond PLUS color change adhesive resin was used to bond the orthodontic ceramic brackets according to the manufacturer instruction. Schematic illustration of the sample preparation and the experimental design are described in Figure 1.

### 2.4. CP-OCT Assessment

Crosspolarization optical coherence tomography (CP-OCT; IVS300, Santec, Japan) was used in this study. Technological specifications of the utilized CP-OCT are described in Table 2. This noninvasive imaging system uses a continuous wavelength diode laser centered near 1310 nm with a 30 kHz scanning rate. CP-OCT.

All specimens assigned to the shear bond strength tests were examined under the CP-OCT system after the bonding procedures to exclude misplaced brackets and defective bonded specimens; this was followed by examining the specimens by the CP-OCT to determine the quality of the orthodontic ceramic bracket-tooth interface.

Due to the different absorption of the OCT rays in the ceramic brackets, the examined interface between the ceramic brackets and enamel appeared on two levels. For simplification of the obtained results, the authors decided to conduct an experiment in which Transbond XT resin was condensed in Teflon 2 mm diameter×2 mm height mold and light cured to obtain 15 composite cylinders. The composite cylinders were divided into three groups and were bonded to lingual surfaces utilizing the same condition of the three experimental groups. These group of samples had their enamel-ceramic brackets interfaces on one level to elaborate the capability of examining interface and in the same time clarify the effect of orthodontic bracket material on the CP-OCT observation.

To conduct the CP-OCT test, each bonded specimen was placed on a micrometer stage with a 3D axis (*x*, *y*, *z*), and the OCT probe was positioned perpendicularly on the buccal surface to scan the bonded interface across the bracket. Consecutive scans were accomplished at 500 *μ*m intervals. The size of each image was 500 × 924 pixels corresponding to 5 mm × 8.2 mm (*x*, *z*).

### 2.5. Shear Bond Strength Test (SBS)

15 bonded specimens from each group were mounted on a universal testing machine (ElectroPlus E1000, Instron, Canton, MA, USA) and subjected to a shear force at the interface between the enamel and the orthodontic bracket at a crosshead speed of 0.5 mm/minute.

### 2.6. Nanoleakage Test

The nanoleakage solution was prepared in a dark room by dissolving 25 g of silver nitrate crystals (Sigma Chemical Co., St. Louis, MO, USA) in 25 ml of distilled water [13]. Dilution of ammonium hydroxide (Sigma Chemical Co., St. Louis, MO, USA) was conducted under proper suction in a specialized hood to obtain 28% ammonium hydroxide solution. The black silver nitrate solution was titrated by 28% ammonium hydroxide that was stirred using magnetic stirrer until it became clear. 50 ml of distilled water was added to obtain a 50% wt ammoniacal silver nitrate pH = 9.5 solution [13, 14]. All measures were carried out to avoid hand or surface contamination by the silver nitrate solution due to its staining properties. 10 bonded specimens from each group were vertically cross-sectioned with a diamond saw under water cooling through the bracket-enamel interface. The central slab was chosen from each tooth, forming a total of 10 specimens per group, *n* = 10. Bonded slabs were ground and polished using wet #1000 silicon carbide paper, then coated with two layers of fast-drying nail varnish applied up to 1 mm from the bonded interface [13, 14]. The specimens were stored in ammoniacal silver nitrate in total darkness for 18 h, rinsed thoroughly, and immersed in photo developing solution (Kodak, NY, USA) for 6 h under fluorescent light to reduce silver ions into metallic silver [13]. The silver-stained resin-bonded specimens were lightly polished to remove the superficial silver remnants [8, 13, 14], followed by drying the specimens and gold sputter coating. The specimens were observed using SEM/EDS (JCM-6000, NeoScope, JEOL, Tokyo, Japan), and line scans were examined across the resin-enamel [8, 13, 14].

### 2.7. Attenuated Total Reflectance Fourier Transform Infrared Spectroscopy (FTIR/ATR) Degree of Conversion Analysis

**Figure 2:**
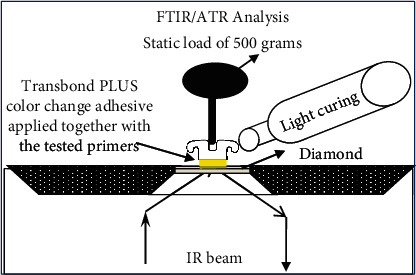
FTIR spectroscopy was performed using the ATR accessory and examination crystal. All measurements were obtained under the following conditions: a resolution of 4 cm^−1^ and four internal scans per reading. FTIR: Fourier-transform infrared; ATR: attenuated total reflectance. Crystal: plate of zinc selenite crystal.

Transbond PLUS Color Change Adhesive (3 M Unitek) was applied to the base of 30 metallic brackets. A thin layer of the unpolymerized moisture insensitive primer was applied in groups MIPD and MIPW, while Transbond XT primer was applied in group XTP *n* = 10. The brackets in group MIPW had 3 *μ*l of distilled water applied on top of the FTIR examination window using a microbrush [10]. All specimens were placed under a static load of 100 grams on the FTIR/ATR examination window [15] as shown in Figure 2 (Thermo Scientific Nicolet iS5 FTIR Spectrometer; Thermo Fisher Scientific, Waltham, MA, USA).

The specimens were light cured by a LED unit (Ortholux; 3 M Unitek) with an output of 1,600 mW/cm^2^. The light curing unit was calibrated using the Demetron™ LED radiometer. The LED unit was slightly touching the bracket, and the curing time was 3 seconds mesial and 3 seconds distal to the brackets.

All FTIR/ATR measurements were obtained under the following conditions: a resolution of 4 cm^−1^ and four internal scans per reading [15]. The uncured resin in each group served as the control for the cured resin.

The spectra of the monomers and their respective polymers were compared to determine the conversion rate of the double bonds into simple carbon bonds. The peaks were measured at the frequencies of 1,715/cm^−1^ (corresponding to the aromatic ring bonds) and 1,637/cm^−1^ (corresponding to the bonds between carbons of the methacrylate groups) [15]. The following formula was used to calculate the conversion rate of the double carbon bonds into simple bonds.

(1) $$\begin{array}{c} \%\text{Conversion}=100\times 1-\frac{\text{Polymer}\left(\text{C}=\text{C}\right)\times \text{monomer}\left(\text{C}-\text{C}\right)}{\text{Monomer}\left(\text{C}=\text{C}\right)\times \text{polymer}\left(\text{C}-\text{C}\right)} \end{array}$$

### 2.8. Statistical Analysis

Shear bond strength and the degree of conversion results were analyzed using one-way ANOVA, *p* ≤ 0.05. Evaluation of nanoleakage locations was carried out through analysis of the obtained images. The infiltration of silver nitrate into the interface was evaluated as follows: enamel-hybrid layer, adhesive–hybrid interface, and adhesive layer were evaluated and graded as (no) (no leakage, score 0), (slight) (slight leakage, score 1), and (distinct) (distinct leakage, score 2). Analysis was carried out using the Kruskal–Wallis test *p* ≤ 0.05 [[8]], and the differences were considered statistically significant at the level of 0.05 5% (SPSS v24, IBM, Armonk, US).

## 3. Results

### 3.1. CP-OCT Results

**Figure 3:**
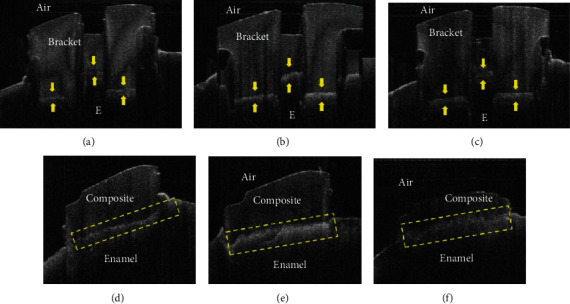
Representative OCT B-scans for the tested groups. (a) Weak backscattered reflections (arrow) at the interface between bracket and enamel can be detected in the XTD group. The bonded interface appeared on two levels due to the difference in thickness of the ceramic bracket parts and laser back schatter in these areas. (d) Weak backscattered reflection dotted box at the interface between the composite cylinder and enamel can be detected in XTD group. The bonded interface appeared on one line due to using composite cylinders for bonding. (b) Strong backscattered reflection (arrows) at the interface between bracket and enamel can be detected in the MIPW group. The bonded interface appeared on two levels due to difference in thickness of the ceramic brackets parts and laser back scatter in these areas. (e) Strong backscattered reflection dotted box at the interface between the composite cylinder and enamel can be detected in the XTD group. The bonded interface appeared on one line due to using composite cylinders for bonding. (c) Weak backscattered reflection (arrow) at the interface between bracket and enamel can be detected in the MIPD group. The bonded interface appeared on two levels due to difference in thickness of the ceramic brackets parts and laser back scatter in these areas. (f) Weak backscattered reflection dotted box at the interface between the composite cylinder and enamel can be detected in the MIPD group. The bonded interface appeared on one line due to using composite cylinders for bonding.

Figures 3(a)–3(c) demonstrate the OCT B-scans for the bonded ceramic brackets assigned to the three experimental groups. Weak backscattered reflection was associated with the images obtained from the XTD group. The backscatter pattern detected in the MIPD group resembled the results in the XTD group. The MIPW wet group was associated with stronger back scatter reflection when compared to the XTD and MIPD groups. Figures 3(d)–3(f) demonstrate the OCT B-scans results of bonding the resin cylindrical specimens to the tooth structure, which yielded the same results obtained for the ceramic bracket bonded specimens.

### 3.2. Shear Bond Strength (SBS) the Abbreviation Is Not Written before

The obtained results of the SBS test were presented in (Table 2). One way ANOVA showed a significant decrease in SBS for MIPW when compared to MIPD and XTP groups (*p* > 0.05) while there was no significant difference in shear bond strength between MIPD and XTP (*p* > 0.05).

### 3.3. Nanoleakage Assessment

**Figure 4:**
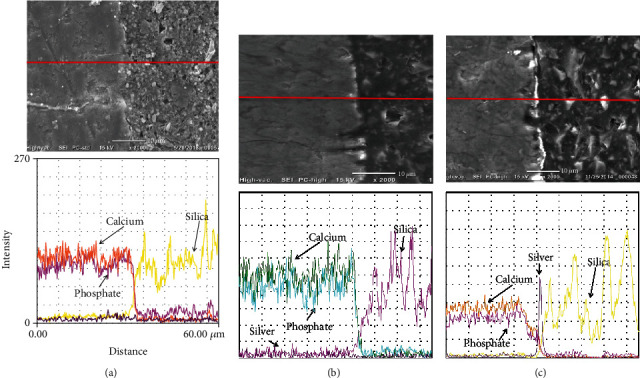
SEM-EDS pictures for the experimental groups. (a) XTP group interface was completely free from any silver nitrate particles. (b) The MIPD group showing minimal infiltration of silver nitrate particles that were below the level of detection by EDS line analysis. (c) The MIPW group interface showed heavy silver nitrate deposits that were observed in all examined regions along the enamel-resin interface.

SEM-EDS pictures' analyses shown in Figure 4(a) demonstrated that the group XTP interface was completely free from any silver nitrate particles. MIPD shown in Figure 4(b) illustrated minimal infiltration of silver nitrate particles that were below the level of detection by EDS line analysis. Heavy silver nitrate deposits were observed in all examined regions along the enamel-resin interface in the MIPW group as shown in Figure 4(c). Statistical analysis showed that there was a significant number of samples showing nanoleakage at various regions of the bonded interface in the MIPW group, *p* = 000 (Table 3).

### 3.4. Degree of Conversion

The degree of conversion results (Table 3) for the specimens in group XTP cured by the Ortholux light recorded the significant highest degree of conversion among the tested groups *p* = 000, followed by the specimens in group MIPD, while the least degree of conversion results was recorded in the MIPW *p* = 000.

## 4. Discussion

This experiment shed light on the sensitivity of the orthodontic bonding procedures and the effect of the bonding substrate condition on the success of this step. The null hypotheses adopted in this experiment were rejected because the CP-OCT technique successfully observed the interfacial defects between the ceramic brackets and the tooth structure; moreover, the increased moisture of the enamel surface negatively affected the MIP bond strength to enamel and increased the nanoleakage expression in the resin-enamel interface.

In this study, CP-OCT showed distinct variation in light reflectance when examining the bonding interface between the ceramic bracket and the enamel surfaces in the three experimental groups. The high backscattered reflection was associated with the MIPW group, and it may be attributed to the difference in the refractive indices at the interface. Since MIPW specimens were not completely dried, contamination of the interface with the residue of water would create some microspaces that would interfere with light penetration causing light scattering in the form of diffuse reflection or so-called “Fresnel phenomenon” [16]. In addition, there is a huge difference between the vapor pressure of ethanol and water which are equal to 5.95 kPa and 2.3 kPa (at 20°C), respectively [16]. The amount of ethanol in the formula of MIIP is within 40%, adding this to HEMA inclusion in this resin would retain some amount of water residue that would interfere with the evaporation process and consequently will negatively affect the polymerization. Water and solvent entrapment along with poor resin polymerization has caused this diffuse reflection in MIPW, unlike MIPD and XTP.

The CP-OCT results suggested that the 2-5 sec (recommended by the manufacturer) of air blowing of MIP after its application on the wet enamel surface was insufficient to completely evaporate the solvent and the excess water. The aforementioned suggestion was confirmed by the infiltration of the bonded interface with silver nitrate and decrease in the degree of conversion of the resin polymerization and deterioration of the shear bond strength observed when enamel surface was moist.

Failure of the solvent contained in the MIP to evaporate the moisture on enamel might have resulted in the absorption of this moisture by HEMA in the uncured MIP primer by water resulting in dilution of the monomers to the extent that polymerization of the primer, and the Transbond XT plus might have been hindered [10, 17, 18]. The aforementioned hypothesis was confirmed by the degree of conversion experiment, which demonstrated a severe depletion in the degree of conversion for the MIP primer when being exposed to moisture condition, agreeing with previous research [10] which showed that C=C stretching vibration (1638 cm^–1^) of the MIP exposed to moisture was not changed after being exposed to light curing polymerization, and that large portion of the primer remained liquid [10].

Moreover, previous research showed that HEMA fixed in a polymer chain after polymerization will still exhibit hydrophilic properties and will lead to water uptake with consequent swelling, discoloration [19] and may even diffuse out of its matrix to the surrounding tissues [20] with a possible contribution to allergic reactions [21]. It was previously demonstrated that HEMA lowers the vapor pressure of water [22] and probably also of alcohol [22], which may explain why the high percentage of ethanol present in the MIP failed in evaporating all water that was on the enamel surface. Moreover, the vapor pressure of water (2.3 kPa at 20°C) is lower than ethyl alcohol (5.8 kPa at 20°C), which means that water requires a prolonged time of air blowing to evaporate when the ethyl alcohol is used as a solvent in the bonding system [22].

On the other hand, previous research showed that HEMA is vulnerable to hydrolysis [23–25]; thus, it may be speculated that the nanoleakage [1] defects observed in the MIP applied on wet enamel surface may develop into microleakage [3] and increase the chance of caries incidence under the cemented orthodontic brackets.

Our results confirmed previous literature [7, 9–11] that reported the deterioration of the shear bond strength of the MIP when bonded to moist enamel or contaminated by blood or saliva; however, observation of the sealing ability of the interface in the current experiment shed light on a possible mechanism that explains the decrease of the bond strength of MIP to moist enamel.

Although there was a significant decrease in the bond strength of MIP to moist enamel, however, the MIP bond strength values were still within the acceptable range for clinical bonding of orthodontic brackets [26, 27].

It is of prime importance to state that any results obtained from the current in vitro experiment should be interpreted cautiously. This is due to the fact that all attempts were done to simulate the clinical situation in the current experiment; however, using the MIP in the oral environment may exert more challenges like the continuous acidic attacks exerted by the bacterial biofilm that is abundant around the orthodontic brackets [2, 28]. Moreover, the variable thermal changes [29] in the oral cavity and the various occlusal stresses exerted on the brackets bonded to the malaligned teeth may aggravate the deterioration of the bond interface inside the patients' oral cavity.

Further studies are required to quantify the degree of reflectance and directly correlate it to the interfacial defects observed in the orthodontic ceramic-enamel interface.

## 5. Conclusions

It is of prime importance to obtain a clean, dry enamel surface prior to orthodontic bonding procedures to obtain reliable results regarding the bond strength and sealing ability of the bonding orthodontic resin under orthodontic brackets. CP-OCT may be a valuable tool to examine and follow up the quality of the bonded orthodontic ceramic bracket-enamel interface in clinical situations.

## Figures and Tables

**Table 1:** Composition of the materials used in the study.

Material (manufacturer) code	Ingredients	Lot No.
Transbond XT light cure adhesive primer (3 M Unitek, USA) XTP	(i) Bisphenol A diglycidyl ether dimethacrylate (45–55%Wt) (ii) Triethylene glycol dimethacrylate(45–55%Wt)	N611932
Transbond MIP moisture insensitive (3 M Unitek, USA) MIP	(i) Ethyl alcohol (30–40% Wt) (ii) Bisphenol A diglycidyl ether dimethacrylate (15-25% Wt) (iii) 2-Hydroxyethyl methacrylate (10-20% Wt) (vi) 2-Hydroxy-1,3-dimethacryloxypropane (5 15% Wt) (v) Copolymer of itaconic and acrylic acid (5-15% Wt) (vi) Diurethane dimethacrylate (1-10% Wt)	N141377
Transbond PLUS color change adhesive (3 M Unitek, USA)	(i) Silane treated glass (35–45%Wt) (ii) Silane treated quartz (35–45%Wt) (iii) 1,2,3-Propanetricarboxylic acid, 2-hydroxy-, reaction products with 2-isocyanatoethyl methacrylate (5-15%Wt) (iv) Polyethylene glycol dimethacrylate (PEGDMA) (5–15%Wt) (v) Bisphenol A diglycidyl ether dimethacrylate (BISGMA) <2Wt (vi) Silane treated silica <2Wt (vii) Diphenyliodonium hexafluorophosphate <1 Wt	N576253

**Table 2:** Means and standard deviation for shear bond strength and % degree of conversion results. Similar superscripts in horizontal rows are not statistically significant, *p* > 0.05.

	XTPD (Transbond XT, dry)	MIPD (moisture insensitive, dry)	MIPW (moisture insensitive, wet)
Shear bond strength	21.34 ± 3.81^a^	17.9 ± 6.69^a^	10.74 ± 2.96^b^
%degree of conversion	67.8 ± 6.8^a^	49.8 + 7.3^b^	31.3 + 3.8^c^

**Table 3:** Evaluation of nanoleakage location.

	Resin			Resin-hybrid layer interface			Hybrid		
	No	Slight	Distinct	No	Slight	Distinct	No	Slight	Distinct
XTD	10	0	0	10	0	0	10	0	0
MIPD	10	0	0	10	0	0	8	2	0
MIPW	1	0	9	1	1	8	0	0	10

*n* = 10 no: no nanoleakage; slight: slight nanoleakage; distinct: distinct nanoleakage.

## Data Availability

All data were supplied in the submitted manuscript.
